# Stressful life events in electronic health records: a scoping review

**DOI:** 10.21203/rs.3.rs-3458708/v1

**Published:** 2023-10-20

**Authors:** Dmitry Scherbakov, Abolfazl Mollalo, Leslie Lenert

**Affiliations:** Biomedical Informatics Center, Department of Public Health Sciences, Medical University of South Carolina; Biomedical Informatics Center, Department of Public Health Sciences, Medical University of South Carolina; Biomedical Informatics Center, Department of Public Health Sciences, Medical University of South Carolina

**Keywords:** EHR, electronic health records, life change events, negative life events, social determinants of health, stressful life events

## Abstract

**Objective.:**

Stressful life events, such as going through divorce, can have an important impact on human health. However, there are challenges in capturing these events in electronic health records (EHR). We conducted a scoping review aimed to answer two major questions: how stressful life events are documented in EHR and how they are utilized in research and clinical care.

**Materials and Methods.:**

Three online databases (EBSCOhost platform, PubMed, and Scopus) were searched to identify papers that included information on stressful life events in EHR; paper titles and abstracts were reviewed for relevance by two independent reviewers.

**Results.:**

527 unique papers were retrieved, and of these 60 were eligible for data extraction. Most articles (n=24, 40%) were focused on the statistical association between one or several stressful life events and health outcomes, followed by clinical utility (n=14, 23.3%), extraction of events from free-text notes (n=8, 13.3%), discussing privacy and other issues of storing life events (n=5, 8.3%), and new EHR features related to life events (n=4, 6.7%). The most frequently mentioned stressful life events in the publications were child abuse/neglect, arrest/legal issues, divorce/relationship breakup. Half of the papers (n=7) that analyzed clinical utility were focused on decision support systems for child abuse and neglect, while the other half (n=7) were discussing clinical interventions related to social determinants of health in general.

**Discussion and Conclusions.:**

Few studies are available on the prevalence and use of stressful life events in EHR reflecting challenges in screening and storage of stressful life events.

## Introduction

Stressful events that individuals experience during life-course have a wide range of consequences for both mental and physical health [1–3]. At the same time, electronic health records (EHR) often lack information about these events [4]. Even so, care from a whole person should include support for these events within clinical encounters.

By stressful life events in this study, we mean highly stressful events that a person views as undesirable and possibly traumatic. Common stressful events include going through abuse, loss of financial means to support oneself, and death of relatives or intimate partner [5]. A well-established reference collection of such events with an added scale of significance of each event is called the ‘Social Readjustment Rating Scale’, introduced in 1967 and later revised by several authors [6, 7]. It is worth noting that the original scale also included common life events such as getting married, starting school, taking out a mortgage or even the consequences of minor violations of the law.

In the context of public health, stressful life events can be viewed as a major personal shock experienced in one of the domains of social determinants of health (SDOH): education, employment, healthcare, social support, neighborhood and living environment [8]. However, life events are personal psychosocial events that can be potentially missed from an SDOH domain documentation perspective. To give a concrete example, the Committee on the Recommended Social and Behavioral Domains and Measures for Electronic Health Records recommends assessment of whether a patient is employed or unemployed/laid-off, but a related stressful event – being fired or laid-off from a job – falls in the recommended measures under the category of stress. The Committee proposed to assess stress using a single question which, arguably, is better suited for measuring current stress than stressful life events (“Stress means a situation in which a person feels tense, restless, nervous, or anxious, or is unable to sleep at night because his/her mind is troubled all the time. Do you feel this kind of stress these days?”) [9]. The experience of unexpected job loss is distinct from daily stress and from the state of being unemployed and its stressful effects might last long after employment is regained.

There are other reasons to consider life events separately from SDOH or as a distinctive group or dimension inside SDOH. Information about such events, in our opinion, doesn’t have a commonly mentioned limitation of SDOH as part of EHR – the practical inability to act upon them in a clinical setting [10, 11]. On the contrary, traumatic events may have a more direct clinical utility. For example, care providers may consider them when assessing symptoms or refer patients to mental health specialists. Considering such life events can be critical in proactive suicide detection [12]. On the other hand, it is worth considering life events occurring in the wider social network of a patient. For instance, a spouse experiencing job loss also can be a perpetrator of family violence [13].

Some stressful personal events, such as the death of a beloved family pet, are difficult to express within the SDOH framework; others such as experiencing gender dysphoria or being arrested, tend to be missed because they are considered “rare events” [9, 14]. Arguably, life events as individual experiences can be better approached using an anthropological paradigm [15]. Nevertheless, a range of stressful life events is incorporated as part of SDOH codes in the ICD-10 edition [16]. This approach, while having limitations noted above, is beneficial for creating a more coherent psychosocial portrait of the patients [17].

The incorporation of SDOH into EHR is rapidly evolving [11, 18], and stressful events, similar to SDOH factors, may be underrepresented in EHR. Practical challenges surround collection of information related to ICD-10 Z-codes [19]. Several researchers noted that much more information about SDOH can be retrieved from clinical notes in EHR compared to structured EHR fields [20, 21]. On the other hand, research shows that clinical notes such as social history usually do not provide a comprehensive view of the social situation of a patient and tend to miss significant life experiences [22].

To our knowledge, no previous study has examined how stressful life events are stored and used in EHR. Thus, the goal of this scoping review is to review and summarize the literature on (1) how well stressful life events are captured in EHR, (2) whether they are stored in structured or unstructured form, (3) what tools are proposed to extract such events from EHR, and (4) how such life events from EHR are used in clinical workflows and further research on patient risk factors and outcomes.

## Methods

The research protocol for this study was created and revised following the recommendation of Preferred Reporting Items for Systematic Reviews and Meta-analysis (PRISMA) [23].

To be included in the review, references had to focus on EHR and either discuss stressful life events in general or focus specifically on some types of the events, such as the death of a relative or marital dissolution. All English-language publications were considered, including dissertations and conference abstracts. Citations were excluded from full-text screening for several reasons:

1. The citation was focused on daily stress rather than stressful life event;
2. The citation discussed normative life course events such as employment, unemployment, marriage, childbirth, unless there is an indication that they are treated as stressful or traumatic life events (and not as demographic status variables);
3. Physical conditions such as diseases, injuries, and pregnancy were not considered as stressful life events, unless they were explicitly considered as such.

The first search was conducted in June of 2023, using three databases: PubMed, Scopus, and all sources in EBSCOhost platform. The list of search terms was compiled based on the Social Readjustment Scale and Adolescent Life Change Event Scale [7, 24]. Search query strategy for one of the databases developed with the assistance of an experienced librarian (Supplementary Appendix S1). In August of 2023, an additional search was initiated with the same databases, but other set of search terms to fetch more articles reflecting the clinical utility of stressful life events in EHR (Supplementary Appendix S2). All search results were exported to Covidence online software, where the remaining review and extraction took place.

The screening was independently performed by two authors (DS and AM), and the data charting form was completed by one author (DS). Disagreements were resolved by reaching a consensus during discussions.

The data charting form was designed to answer the following research questions: how stressful life events are stored in EHR (structured, unstructured, or linked data); if they were not stored in EHR where they were sourced from (survey, external dataset); what is the focus of the publication (finding a statistical association, extracting of life events from unstructured data, discussing issues of capturing life events, clinical utility); what types of events were considered by authors. Additionally, for studies focused on the statistical association between life events and other variables: what was the nature of outcome variables (physical or mental health area). For citations focused on clinical utility details on clinical workflows were extracted. In addition, the region of each publication was identified.

## Results

**Figure 1:**
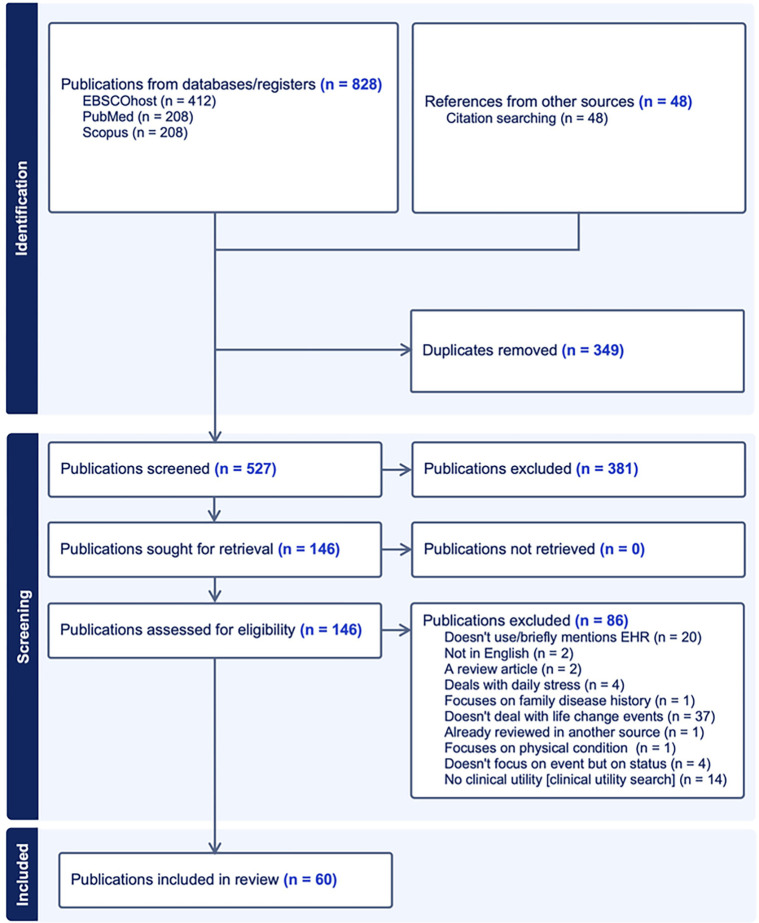
Flow diagram of citation selection process

Figure 1 illustrates the PRISMA article selection process. Initially, 527 unique citations were identified from searches of electronic databases and reviewing article references (snowballing). After the title and the abstract screening, 381 were excluded, with 146 full-text publications to be retrieved and assessed for eligibility. Of these, 86 did not meet the inclusion criteria. The remaining 60 citations were considered eligible for inclusion in this review.

**Figure 2:**
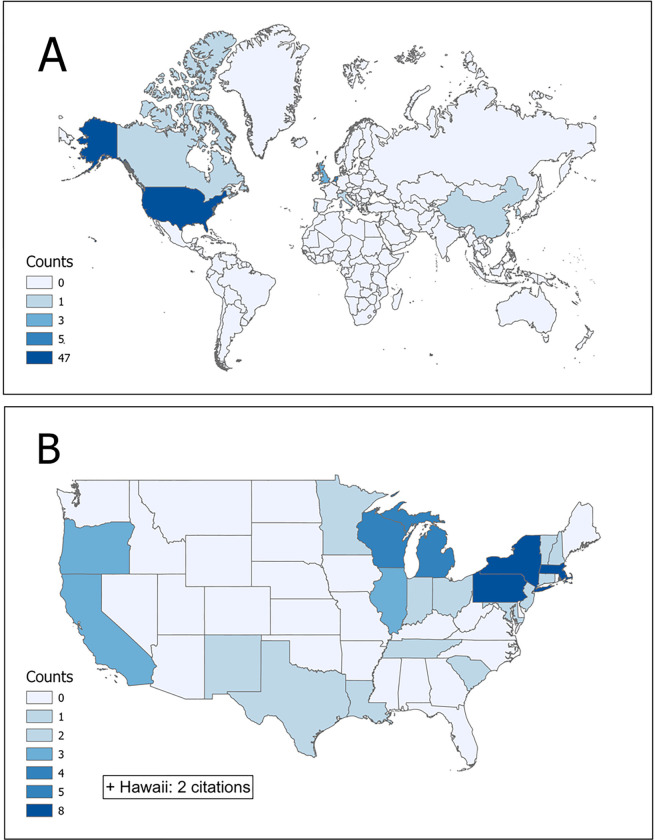
Citations by place of origin. A: By country; B: By US state.

Most citations focusing on stressful life events and EHR were coming from the US (n=47, 78.3%), Netherlands (n=5, 8.3%) and UK (n=3, 5%). Canada, China, Italy, Portugal, and South Korea were represented by one publication. Reference characteristics by country of origin and US states statistics are provided in Figure 2A.

Most citations can be categorized into five following areas by their focus:

1. Finding statistical dependency, in which one of the variables is stressful life event(s) (n=24, 40%);
2. Clinical utility of stressful life events in EHR (n=14, 23.3%);
3. Extracting events from EHR (n=8, 13.3%);
4. Discussing issues of storing life events in EHR (n=5, 8.3%), such as privacy;
5. Proposing new EHR design, template, or feature to facilitate stressful life events capturing (n=4, 6.7%).

**Figure 3:**
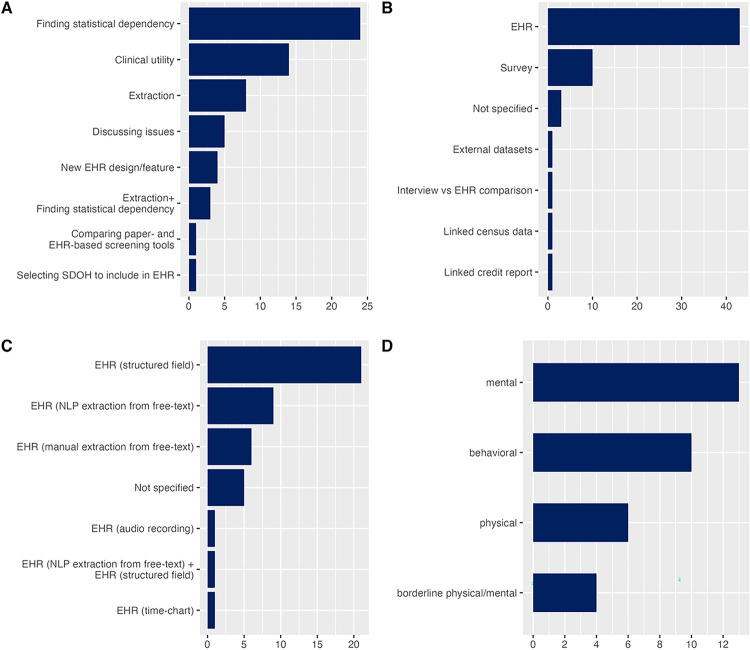
A: Citations by area of focus; B: Citations classified by source of stressful life events; C: Citations by EHR data types and methods of extraction; D: Outcome variables in statistical studies by their area.

The remaining 5 publications were focused as follows. Three citations [25–27] spanned two areas in a single study: finding statistical dependency and extraction. One study [28] compared paper-based and EHR-based screening tools and found that capturing adverse life events improved significantly when using specialized tools in EHR: 77% of EHR had information on such events compared to 33% when using older paper-based tools. One more citation [9] is the already mentioned work of the Committee on the Recommended Social and Behavioral Domains and Measures for Electronic Health Records, which debated what measures should be considered for stressful life events and decided that a general measure of stress would be sufficient. Figure 3A depicts the focus areas of all included articles.

Most of the citations (n=43, 71.7%) discussed or utilized life events stored in various formats in EHR. However, a significant number of studies used events obtained via surveys conducted specifically for the study (n=10, 16.7%) – these were statistical studies, and EHR was most often used as a source of variables related to health outcomes. One more citation utilized the interview format for events identification and compared them to what is documented in EHR and found that health records “generally failed to include social contexts salient to patients” [29]. Another publication linked a credit history report to identify serious financial events in patients’ lives, such as bankruptcy [30]. One citation [31] discussed issues of using external data sources linked to EHR for capturing “financial, legal, life event and sociodemographic data” to improve suicide screening models. One more study [32] investigated the impact of the 2008 economic crisis on different health outcomes using the unemployment data from the census. Figure 3B demonstrates breakdown of the articles by source of stressful life events.

The publications that were focused on EHR as a source of stressful life events used structured data on events in more instances (n=22, 56.4%) than unstructured data (n=17, 43.6%). One additional paper [33] discussed a time chart feature inside EHR showing correlations between life events and body mass index. In citations using unstructured data natural language processing (NLP) was the most popular method for extraction (n=10, 58.8%), followed by manual abstraction (n=6, 35.2%), however, audio recording was used in one citation [34]. Figure 3C illustrates the data types for stressful life events in EHR.

In studies that were focused on finding statistical dependency the health outcome under question was most often in the domain of mental health (n=13, 39.4%), such as diagnosis of post-traumatic stress disorder, and anxiety; followed by behavioral outcome variables related to violent and self-harmful behavior (n=10, 30.3%), and physical health outcomes (n=6, 18.2%). Few outcome variables (n=4, 21.1%) were in the borderline area spanning across physical and mental health, such as in the case of chronic pain. Figure 3D summarizes observed outcome variables.

**Figure 4:**
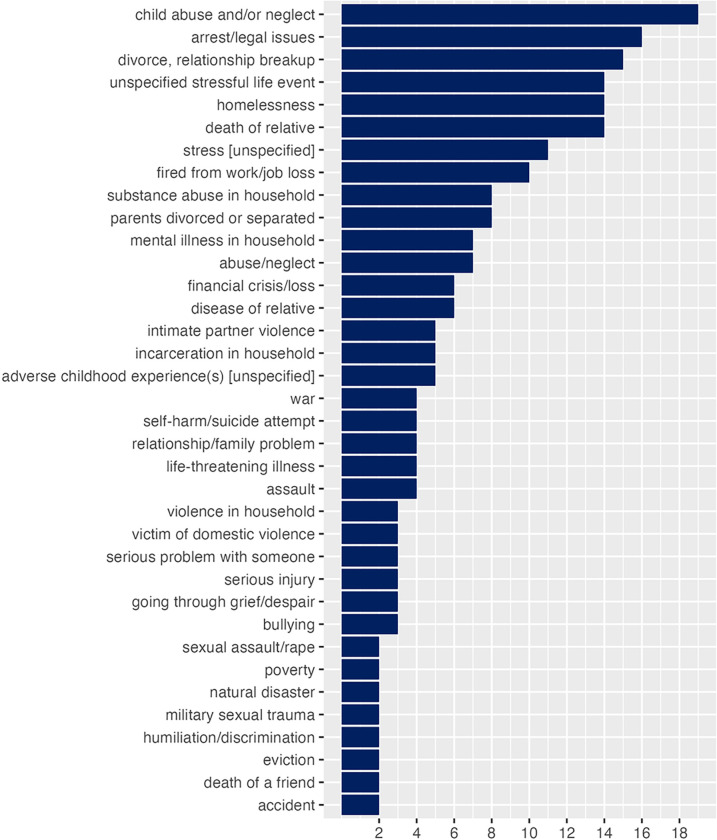
Stressful life events by frequency of mention in the citations (excluding events with one mention only)

There were recurring stressful life events seen across multiple citations, such as child abuse and/or neglect, arrest/legal issue, divorce or relationship breakup, death of relative, loss of job, homelessness. Different types of stressful life events which were mentioned by two or more studies are presented in Figure 4. A sizable number of events were mentioned by one study only: abandonment by parent, captivity, child marriage, disappearance of family member, forced marriage, genital mutilation/cutting, life-threatening event, miscarriage, parental behavioral problems, unspecified psychological trauma, sex trafficking in childhood, sexual exploitation, something valuable lost or stolen, starvation, terrorism, taking drugs as a child, unwanted pregnancy, victimization experience, witnessing somebody’s injury or death.

Child abuse and/or neglect was the most common type of stressful life event mentioned in citations (n=19, 31.7%). A sizable number of publications (n=14, 23.3%) considered a serious life event but did not specify what event it was. An example of this approach is a survey question “After the age of 17, did you experience any major upheaval that you think may have shaped your life or personality significantly?” [35]. Unspecified stress was used in a sizable portion of citations (n=11, 18.3%), a common question to capture it was “Stress means a situation in which a person feels tense, restless, nervous, or anxious, or is unable to sleep at night because his/her mind is troubled all the time. Do you feel this kind of stress these days?”. This question is used in various popular screening tools such as PRAPARE [36–38].

Among the articles that analyzed clinical utility, a half (n=7) were focused on child abuse and neglect. These publications discussed several clinical processes which are triggered when such events are detected: alert in EHR, starting an abuse-related order set, and reporting to child protection agencies. The other half (n=7) touched on the topic of possible clinical workflow alterations based on detected SDOH categories, among which some stressful life events were mentioned: homelessness, domestic violence. However, because these publications considered broad SDOH domains, we couldn’t extract specific workflows that would be related to stressful life events. Some of the general SDOH-related clinical interventions discussed in these papers included: referral to trauma recovery [39], sending educational materials to patient [39], alerts and triggers [36, 40], panel management [36], automatic and manual referrals to third party agencies [36, 38, 41], risk adjustments [42], modification of clinical practices [36, 41], adjusted provider recommendations [36, 41].

Individual citations characteristics in table format are provided in Supplementary Appendix S3. For some of the citations the table also includes additional extracted characteristics, such as dependent and independent variables in statistical studies, types of surveys used to capture events, NLP tools used, relevant information from results, discussion and conclusion sections of reviewed citations.

## Discussion

Stressful life events are a distinct concept from SDOH, being more intrapersonal or interpersonal experiences than external factors. In this review, we found a limited number of citations related to stressful life events and EHR documentation, suggesting this is an area of research with substantial gaps. The obvious explanation for the gaps is that information about life events is not captured adequately in EHR. The improvement of existing NLP tools for parsing clinical notes to include a broader spectrum of life events could facilitate the research while existing SDOH screening tools could be modified to foster collection of such events.

This study showed that there is significant subjectivity in defining stressful life events which may prevent collection of such events using predefined lists. Previous research shows that even life events which head the list of the Social Readjustment Scale, such as divorce, have a significant interindividual variability and can improve well-being in some instances [80, 81]. To address the problem of screening for stressful life events, it may be beneficial to assess information about recent (12 months or less) life events using a single question on adverse life events with a more detailed approach for distant life events [4].

Many citations reviewed relied on a single question for stress assessment proposed by the Committee on the Recommended Social and Behavioral Domains and Measures for Electronic Health Records [9]. However, when reviewing these publications, it was difficult to differentiate daily stress from stressful life events, which this question is supposed to capture. In our opinion, alternative screening approaches need to be considered. For instance, artificial intelligence technologies like chatbots could facilitate consistent data collection about life events and other important health-related aspects of people’s lives in a non-intrusive manner. Querying external data sources like population registry, social media, census, police, and financial records for stressful events is feasible, especially if such modules are developed by EHR software vendors. All these approaches require careful workflow planning to ensure that sensitive life details of a patient and his family are protected. One possible solution could be that a particular life event that a person experienced is hidden from most health professionals, and only a general alert flag is set (“Recent negative/traumatic life situation experienced”).

This may prompt a dialog between the care provider and the patient so that the latter can provide more details if they feel inclined to.

Clinical utility of stressful life events was mostly represented by papers on decision support systems for identification of potential child abuse – an area which has a well-established tradition of clinical practice. This suggests that new workflows addressing other types of stressful life events in EHR are yet to be defined.

### Limitations

One of the limitations of this scoping review lies in the definition of stressful life events. Some events mentioned in the studies reviewed, such as household moves and immigration, were considered normative by our team and were excluded from review. More stressful events and situations could be identified by using a broader search strategy and including more types of events or by adding domains of SDOH to the search terms. Another limitation is that we didn’t focus specifically on the types of NLP methods that were used to extract events from EHR, although some information about methods is listed in Supplementary Appendix S3. Comparing a list of methods used could be beneficial in seeing how each type of event can be extracted most efficiently. However, this topic is separate from the importance of stressful life events and their impact. The review does not assess how exactly life events storage was implemented in EHR software as most studies did not include sufficient details on this topic.

## Conclusions

Stressful life events are an important issue that complements work on SDOH. More data is needed on how to best capture these events in patients’ health record and/or to extract such events from text descriptions in clinical notes, and how to best intervene to support patients experiencing stressful events in clinical settings.
